# Temporal associations between depressive features and self-stigma in people with substance use disorders related to heroin, amphetamine, and alcohol use: a cross-lagged analysis

**DOI:** 10.1186/s12888-022-04468-z

**Published:** 2022-12-21

**Authors:** Mohsen Saffari, Kun-Chia Chang, Jung-Sheng Chen, Ching-Wen Chang, I-Hua Chen, Shih-Wei Huang, Chieh-hsiu Liu, Chung-Ying Lin, Marc N. Potenza

**Affiliations:** 1 Health Research Center, Life Style Institute, Baqiyatallah University of Medical Sciences, Tehran, Iran; 2 Health Education Department, Faculty of Health, Baqiyatallah University of Medical Sciences, Tehran, Iran; 3 Department of General Psychiatry, Jianan Psychiatric Center, Ministry of Health and Welfare, Tainan, Taiwan; 4 Department of Psychiatry, National Cheng Kung University Hospital, College of Medicine, National Cheng Kung University, Tainan , Taiwan; 5 Department of Medical Research, E-Da Hospital, Kaohsiung, Taiwan; 6 Graduate Institute of Social Work, National Taiwan Normal University, Taipei, Taiwan; 7 Chinese Academy of Education Big Data, Qufu Normal University, Qufu City, Shandong China; 8 Institute of Environmental Toxin and Emerging Contaminant, Cheng Shiu University, Kaohsiung, 83347 Taiwan; 9 Center for Environmental Toxin and Emerging-Contaminant Research, Cheng Shiu University, Kaohsiung, 83347 Taiwan; 10 Department of Family Medicine, Taoyuan General Hospital, Ministry of Health and Welfare, Taoyuan, Taiwan; 11 Institute of Allied Health Sciences, College of Medicine, National Cheng Kung University, Tainan, Taiwan; 12 Biostatistics Consulting Center, College of Medicine, National Cheng Kung University Hospital, National Cheng Kung University, Tainan, Taiwan; 13 Department of Public Health, College of Medicine, National Cheng Kung University, Tainan, Taiwan; 14 Department of Occupational Therapy, College of Medicine, National Cheng Kung University, Tainan, Taiwan; 15 Department of Psychiatry, Yale School of Medicine, New Haven, CT USA; 16 Connecticut Mental Health Center, New Haven, CT USA; 17 Connecticut Council On Problem Gambling, Wethersfield, CT USA; 18 Child Study Center, Yale School of Medicine, New Haven, CT USA; 19 Department of Neuroscience, Yale University, New Haven, CT USA; 20 Wu Tsai Institute, Yale University, New Haven, CT USA

**Keywords:** Substance-related disorders, Alcohol use disorder, Amphetamine use disorder, Opioid use disorder, Addictive behaviors, Cross-lagged analysis, Depression, Longitudinal study

## Abstract

**Background:**

Depression is a mental health problem and substance use concerns are socially unacceptable behaviors. While depression and substance use may individually impact self-concept and social relationships, their co-occurrence can increase the risk of self-stigmatization. However, there is no evidence regarding how depression and self-stigma may influence each other over time. The aim of the current study was to evaluate the cross-sectional and longitudinal relationships between features of depression and self-stigma in people with substance use disorders.

**Methods:**

Overall, 319 individuals with substance use disorders (273 males) with a mean (± SD) age of 42.2 (± 8.9) years were recruited from a psychiatric center in Taiwan by convenience sampling. They were assessed for features of depression and self-stigma at four times over a period of nine months using the depression subscale of the Depression Anxiety Stress Scales (DASS-21) and Self-Stigma Scale-Short S (SSS-S), respectively. Repeated-measures analyses of variance, Pearson correlations and cross-lagged models using structural equation modeling examined cross-sectional and temporal associations between depression and self-stigma.

**Results:**

Positive cross-sectional associations were found between depressive features and all assessed forms of self-stigma over time (0.13 < r < 0.92). Three models of cross-lagged associations between different forms of self-stigma and depressive features indicated good fit indices (comparative fit index > 0.98). The direction of associations between depressive features towards self-stigma was stronger than the opposite direction.

**Conclusion:**

Positive associations between depressive features and self-stigma were found in people with substance use disorders. Although these associations may be bidirectional longitudinally, the directions from depressive features to self-stigma may be stronger than the reverse directions, suggesting treatment of depression in earlier stages may prevent self-stigmatization and subsequent poor outcomes in people with substance use disorders.

**Supplementary Information:**

The online version contains supplementary material available at 10.1186/s12888-022-04468-z.

## Introduction

Depression is a major mental health problem affecting more than 350 million people worldwide [1], including Taiwan with the prevalence has increased by 25% from 2007 to 2016 [2]. Depression could be influenced by social and economic factors and may negatively impact daily functioning, leading to high personal and societal costs [3] and result in a top factor for healthy life lost [4, 5]. Unfortunately, many people with depression suffer from addictive behaviors, particularly those relating to substance use. The use of substances may constitute a coping mechanism to escape from their symptoms, and this in part explains the close relationships between substance use disorders (SUDs) and depression [6].

SUDs are common among people with various socio-demographic backgrounds [7]. Use of psychoactive substances has been associated with psychological distress and functional disturbances [8]. Heroin and amphetamine are two common substances of abuse in Taiwan [9, 10]. Each may generate different subjective mood-altering experiences, with heroin sometimes being used to reduce stress and anxiety and amphetamines to experience stimulation [11]. However, both heroin use and amphetamine use have been linked to depressive symptomatology [12].

As heroin use and amphetamine use are liked to depressive symptomatology, depression and drug use are often inter-correlated and concurrent [8, 10]. More than two-thirds of people with depression have co-occurring drug use disorders [13]. People with histories of drug use disorders are more likely to experience depression, and the likelihood of drug use disorders in the future is increased among people with depression. A national study in the US general population showed that 40% of individuals with lifetime major depressive disorder concurrently had alcohol use disorder or other drug use disorders. Nevertheless, only a small fraction (about 20%) may seek medical care or treatment [14]. Drug use disorders, similarly to depression, can carry high social costs such as disrupted familial relationships and social isolation, and affected people may experience negative emotions relating to decreased intimacy, physical violence, and psychological stress [7, 8, 15]. Studies suggest that people with depression (versus those without) often experience worse drug use disorders and have poorer prognoses [13, 14]. The co-occurrence of these conditions may increase the risk of suicide, particularly in younger adults [16]. However, individuals with co-occurring depression and drug use disorders may experience benefits in both domains when receiving treatment for one condition or the other [8, 15].

In addition to depression and drug use co-occurring mentioned above, the associations between depression and alcohol use disorders is well-documented [17]. Nearly three million deaths annually may be attributable to alcohol consumption worldwide, and alcohol use may contribute substantially to psychological problems [17, 18]. In particular, affective disorders like depression have been associated with alcohol use disorders [19]. However, causal relationships between these disorders are unclear, and it has been proposed that each disorder may trigger the other. Nevertheless, there are psychosocial issues surrounding both disorders regarding how affected people or others may perceive the conditions [20, 21].

Among the psychosocial issues, a prevalent psychosocial concern in people with SUDs or depression is stigmatization, and this may be manifested as perceived social stigma and self-stigma [12, 22]. The former is the fear related to being discriminated against or enacted stigma that may originate in community beliefs, whereas the latter (i.e., self-stigma) is defined as internalization of general beliefs or stereotypes that the public may have toward people who suffer from health concerns including disabilities or disorders [23]. These types of stigma, specifically self-stigma, are common in people with depression or with SUDs and may promote perceptions regarding personal weakness [24].

Apart from the commonness of self-stigma among people with SUDs, self-stigma may interfere with treatment as well. Self-stigma is a serious barrier for care-seeking because individuals may try to prevent being associated with these conditions or being publicly labeled as depressed or addicted [25]. Furthermore, self-stigma may predict negative consequences such as poor quality of life, non-adherence to treatment, and refusal of social support and help from family or friends, making it a significant correlate of SUDs and depression [12, 23, 26]. Self-stigma is also negatively associated with self-esteem, self-efficacy, self-image and hope, and thus self-stigma may lead to psychiatric problems, including suicidal ideation [22, 25, 27]. Together, these data highlight the importance of considering self-stigma when understanding and treating people with co-occurring SUDs and depression.

However, there is a limited body of research on how self-stigma may develop in people with SUDs and depression. According to Corrigan and colleagues, self-stigma in serious mental problems may develop in three stages of stereotype awareness (cognitive self-stigma), acceptance of stereotype (affective self-stigma), and self-concurrence (behavioral self-stigma) [27]. Specifically, people with mental disorders may first become aware of stereotypes communicated by others, and this awareness may alter their cognitions. Later, they may accept these stereotypes by generating negative attitudes toward themselves. Eventually, they internalize these beliefs and demonstrate behaviors related to self-stigmatization [24].

The associations between self-stigma and depression have been assessed in several cross-sectional studies. For example, Khalid et al. [28] found that self-stigma may result in mild depressive symptoms in people seeking treatment for SUDs. Chang et al. [29] found the co-occurrence of self-stigma and depression as influencing persistence of alcohol consumption. However, there is limited evidence on the relationship between depressive symptoms and self-stigma processes in people with SUDs and how these variables may influence each other in this population. Cross-lagged analysis, as a measure to examine temporal associations between two variables over time, may be an appropriate approach to estimate relationships between depressive features and self-stigma and understand directional influences of these variables in a longitudinal study [30]. Findings from such an approach could suggest possible causal inferences regarding relationships between these variables and help develop strategies to prevent depressive features and self-stigma in people with SUDs. In other words, if the findings show some interaction between the depressive features and self-stigma, early interventions targeting each problem may help prevent the transition to more severe forms of the other problem and could help clarify whether the self-stigma or depression may be a gateway to increased risk of the other problem. Moreover, identification of temporal associations between these variables may help healthcare professionals and researchers address potentially causal relationships between them using experimental designs and could assist with finding more effective treatments and preventive interventions.

Based on the extant literature, the current study hypothesized that 1) the severity of depressive features in people with SUDs would positively associate with self-stigmatization cross-sectionally, 2) each form of self-stigma (cognitive, affective, behavioral) would be associated with severity of depressive features, and 3) the strengths of associations longitudinally going from depressive features to forms of self-stigma would be greater than those in the opposite association, suggesting that depressive features would exacerbate self-stigma. A main aim of the study was to examine the influences of self-stigma and depressive features on each other during four times of assessments over a nine-month period in people with SUDs.

## Methods

### Participants and procedure

Participants were recruited from the outpatient department of the Jianan Psychiatric Center (JPC). The JPC is a primary psychiatric center operating through the Integrated Demonstrative Center of Addiction Treatment Pilot Program (IDCATPP), a program launched in 2018 for Taiwan, including Southern Taiwan. The IDCATPP program was funded by the Ministry of Health and Welfare. Moreover, the IDCATPP was launched to help people with SUDs, and the JPC is providing direct treatment. The inclusion criteria for the participants in the present study were (1) aged 20 years or more; (2) having been diagnosed with a SUD (relating to heroin, amphetamine, or alcohol) according to DSM-5 diagnostic criteria [31] by qualified psychiatrists; and (3) possessing sufficient cognitive abilities to complete the instruments used in the present study. Participants with the following conditions were excluded: (1) diagnosed as having intellectual disabilities; (2) diagnosed as having dementia or schizophrenia; and (3) not having received treatment during the study period. Several psychiatrists screened patients and invited eligible individuals for participation. Moreover, the present sample consisted of 112 (35.1%) individuals with heroin use, 151 (47.3%) with amphetamine use, and 56 (17.5%) with alcohol use. It was unknown if there was polydrug use as this information was not collected. The participants received treatments according to their diagnosis. People with heroin use were treated using methadone maintenance treatment with psychological treatment (i.e., individual or group counseling with the techniques of cognitive behavioral therapy), those with amphetamine use were treated using psychological treatment alone (i.e., individual or group counseling with the techniques of cognitive behavioral therapy), and those with alcohol use were treated using naltrexone, disulfiram or acamprosate combined with psychological treatment (i.e., individual or group counseling with the techniques of cognitive behavioral therapy or motivational interviewing). Therefore, if the participants were in the same diagnostic group, they received similar treatments. In all cases, participants received at least one year of treatment.

After completing baseline assessments, each participant was invited to complete the instruments again every three months for nine months. That is, participants could complete the study instruments from one to four times. Data were collected by two well-trained research assistants when participants were in the JPC for outpatient visits or other clinical services. Every participant signed a written informed consent, and the study was approved by the Institutional Review Boards of the JPC (IRB numbers: 19–034 and 19–054).

### Instruments

#### Depression, Anxiety, Stress Scale – Depression subscale (DASS-21-D)

The DASS-21 is a commonly used instrument for assessing psychological distress. It assesses three types of psychological distress: depression, anxiety, and stress [32]. In the present study, the focus was on depression and only the depression subscale of the DASS-21 (i.e., DASS-21-D) was used. There were seven items in the DASS-21-D and each item was rated on a four-point Likert-like scale, where 0 = did not apply to the respondents at all and 3 = applied to the respondents very much/almost all the time [32]. The score of the DASS-21-D was calculated using the sum score of the seven items multiplied by 2, as instructed by the developers [32], and higher scores reflected more severe depressive features. The psychometric properties of the DASS-21 have been reported to be satisfactory [33–37]; for example, the Cronbach’s α of the DASS-21-D Chinese version ranged from 0.82 to 0.84 [34, 35]. In the present study, the Cronbach’s α of the DASS-21-D was 0.92 (baseline), 0.94 (Time 2), 0.95 (Time 3), and 0.93 (Time 4).

#### Self-Stigma Scale-Short (SSS-S)

The SSS-S is a commonly used instrument for assessing self-stigma. It assesses three types of self-stigma: cognitive (i.e., being aware of self-stigma and agreeing with it), affective (i.e., having emotional reactions due to self-stigma endorsement), and behavioral (i.e., having actions such as social withdrawal due to self-stigma endorsement) [38]. There were three items assessing each type of the self-stigma (i.e., each subscale in the SSS-S), and each item was rated on a four-point Likert-like scale, where 1 = strongly disagree and 4 = strongly agree [38]. The score of each SSS-S subscale was calculated using the average score of the three items with higher scores reflecting higher levels of self-stigma. The psychometric properties of the SSS-S have been reported to be satisfactory [39–41]; for example, the Cronbach’s α of the SSS-S Chinese version ranged from 0.80 to 0.91 [39, 41]. In the present study, the Cronbach’s α of the SSS-S cognitive subscale was 0.84 (baseline), 0.90 (Time 2), 0.88 (Time 3), and 0.92 (Time 4). The Cronbach’s α of the SSS-S affective subscale was 0.74 (baseline), 0.80 (Time 2), 0.77 (Time 3), and 0.83 (Time 4). The Cronbach’s α of the SSS-S behavioral subscale was 0.89 (baseline), 0.90 (Time 2), 0.90 (Time 3), and 0.90 (Time 4).

### Data analysis

**Fig. 1:**
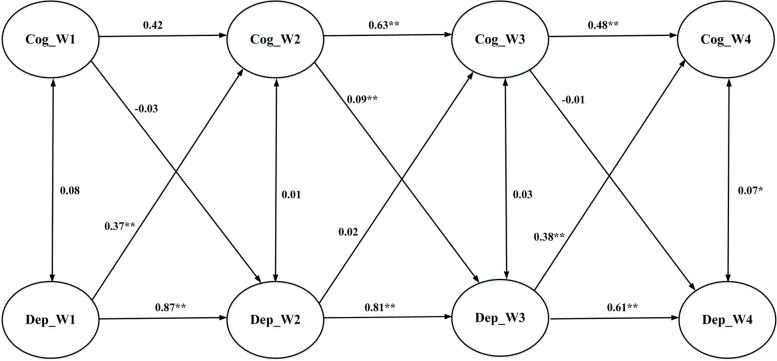
Cross-lagged model examining temporal associations between depressive features and cognitive self-stigma

**Fig. 2:**
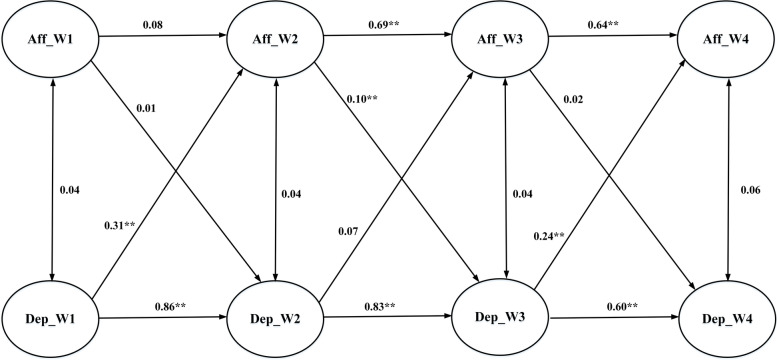
Cross-lagged model examining associations between depressive features and affective self-stigma

**Fig. 3:**
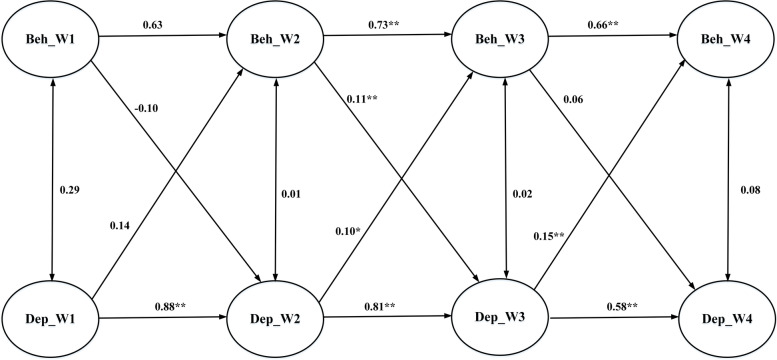
Cross-lagged model examining associations between depressive features and behavioral self-stigma

Participants’ characteristics, including their demographics and depressive and self-stigma scores at baseline, were analyzed using descriptive statistics of means and frequencies. The data were tested for missing values at follow-ups using Little’s Missing Completed at Random test [42], and no patterns of missing not at random were found (χ^2^ = 133.718; df = 128; *p* = 0.35). Multiple imputation was used to impute the missing values for the following inferential data analyses. Repeated measures analyses of variance (ANOVAs) were applied to examine differences in depressive features and self-stigma across time. A Bonferroni adjustment with six tests was used. Therefore, the new significance level was set at *p* < 0.0083 for each comparison in the ANOVAs. Then, Pearson correlations were applied to examine associations between depressive features and each type of self-stigma across four assessment times. Finally, cross-lagged models using structural equation modeling (SEM) were applied to examine temporal associations between depressive features and each type of self-stigma across time. There were three cross-lagged models, with each containing two variables only (depressive features and one type of self-stigma). Therefore, the models were simplified for satisfying the principal of parsimony in the SEM. The first cross-lagged model examined temporal associations between depressive features and cognitive self-stigma (Fig. 1); the second examined associations between depressive features and affective self-stigma (Fig. 2); and the third examined associations between depressive features and behavioral self-stigma (Fig. 3). Diagonally weighted least squares estimators were used for all cross-lagged models. All cross-lagged models were checked for their data-model fit using the following fit indices: comparative fit index (CFI) > 0.9, non-normed fit index (NNFI) > 0.9, relative fit index (RFI) > 0.9, root mean square error of approximation (RMSEA) < 0.08, and standardized root mean square residual (SRMR) < 0.08 [38, 39]. All statistical analyses were performed using IBM SPSS software and LISREL 8.8.

## Results

The participants were 319 patients with SUDs with a mean age of 42.19 (SD = 8.86) years. Most participants were male (*n* = 273; 85.5%). Slightly over half were currently married (*n* = 163; 51.1%), and nearly half only completed compulsory education in Taiwan (i.e., completion of junior high school) or did not complete the compulsory education. Over half of participants (*n* = 209; 65.5%) had a full-time job. Participants with different diagnoses had different depression and self-stigma levels. People with heroin use had the highest levels of self-stigma in all domains (Mean = 2.52 to 2.96), followed by those with amphetamine use (Mean = 2.16 to 2.71) and alcohol use (Mean = 1.76 to 2.21; *p*-values < 0.01) at baseline. Individuals with heroin use had significantly higher levels of depression (Mean = 9.77) than those with amphetamine use (Mean = 6.36) but not those with alcohol use (Mean = 9.51). Table 1 presents participants’ baseline depression and self-stigma information; Table 2 presents relationships between depressive features and self-stigma across time. Significant differences in self-stigma levels were found. The highest levels of all three forms of self-stigma were observed at baseline.

**Table 1:** Participant characteristics (*N* = 319)

Age (years); mean (SD)	42.19 (8.86)
Gender (male); n (%)	273 (85.5%)
Marital status	
Single; n (%)	163 (51.1%)
Married; n (%)	71 (22.3%)
Divorced or separated; n (%)	81 (25.4%)
Others; n (%)	4 (1.2%)
Education	
Elementary school degree; n (%)	24 (7.5%)
Junior high school degree; n (%)	119 (37.3%)
Senior high school degree; n (%)	135 (42.3%)
Undergraduate degree; n (%)	38 (11.9%)
Graduate degree; n (%)	3 (1%)
Occupation	
Full time job; n (%)	209 (65.5%)
Part-time job; n (%)	45 (14.1%)
Unemployment; n (%)	49 (15.4%)
Others; n (%)	16 (5%)

**Table 2:** Self-stigma and depression across time

	Mean (SD)				*F* (*p*-value)	Comparisons using Bonferroni adjustments
	Time 1	Time 2	Time 3	Time 4		
Self-stigma_ Affect	2.34 (0.82)	2.17 (0.75)	2.19 (0.82)	2.19 (0.91)	6.29 (*p* < 0.01)	1 > 2, 1 > 3, 1 > 4
Self-stigma_ Behavior	2.70 (0.91)	2.50 (0.87)	2.49 (0.88)	2.36 (1.07)	18.45 (*p* < 0.01)	1 > 2, 1 > 3, 1 > 4, 2 > 4, 3 > 4
Self-stigma_ Cognition	2.22 (0.89)	2.07 (0.80)	2.14 (0.77)	2.11 (1.01)	3.37 (*p* = 0.02)	1 > 2
Depression	7.94 (10.34)	7.24 (9.83)	7.31 (9.20)	7.06 (8.70)	1.41 (*p* = 0.24)	

Self-stigma assessed using the Self-Stigma Scale-Short form; depression assessed using the Depression subscale of the Depression, Anxiety, Stress Scale (DASS-21). Time 1 = baseline; Time 2 = first follow-up (three months after baseline); Time 3 = second follow-up (six months after baseline); Time 4 = third follow-up (nine months after baseline); Post-hoc comparisons were Bonferroni-corrected

Correlations between each type of self-stigma and depressive features across the four assessment times are presented in Table 3. All correlations were significant, with most having an effect size of small or higher. All proposed crossed-lagged models in examining temporal associations between depressive features and self-stigma had satisfactory fit (Table 4): CFI = 0.981 to 0.999; NNFI = 0.955 to 0.999; RFI = 0.974 to 0.997; RMSEA = 0.000 to 0.080; and SRMR = 0.035 to 0.046. Based on the satisfactory fit, coefficients in the temporal associations were further scrutinized.

**Table 3:** Correlations between self -stigma and depression

		r														
	1	2	3	4	5	6	7	8	9	10	11	12	13	14	15	16
1. Self-stigma_ Affect_T_1_	1															
2.Self-stigma_ Behavior_T_1_	.81	1														
3. Self-stigma_ Cognition_T_1_	.81	.74	1													
4. Depression_T_1_	.40	.34	.42	1												
5. Self-stigma_ Affect_T_2_	.65	.67	.58	.29	1											
6. Self-stigma_ Behavior_T_2_	.64	.69	.56	.29	.86	1										
7. Self-stigma_ Cognition_T_2_	.60	.60	.64	.37	.84	.81	1									
8. Depression_T_2_	.32	.23	.33	.80	.25	.23	.36	1								
9. Self-stigma_ Affect_T_3_	.47	.43	.50	.28	.59	.60	.54	.17	1							
10. Self-stigma_ Behavior_T_3_	.58	.59	.51	.33	.64	.67	.56	.22	.85	1						
11.Self-stigma_ Cognition_T_3_	.44	.43	.54	.31	.60	.56	.61	.21	.87	.78	1					
12.Depression_T_3_	.32	.18	.31	.72	.24	.24	.30	.77	.32	.34	.31	1				
13.Self-stigma_ Affect_T_4_	.56	.51	.44	.41	.61	.58	.56	.33	.58	.65	.54	.36	1			
14.Self-stigma_ Behavior_T_4_	.53	.56	.41	.35	.59	.60	.53	.28	.52	.62	.49	.32	.92	1		
15.Self-stigma_ Cognition_T_4_	.44	.31	.45	.49	.41	.43	.48	.39	.52	.50	.56	.46	.84	.76	1	
16.Depression_T_4_	.21	.14^a^	.13^b^	.44	.23	.25	.29	.48	.14^a^	.19	.13^b^	.57	.19	.16	.23	1

All p-values < 0.01, except for those with a subscript a (*p* = 0.01) or b (*p* = 0.02)

*T*_*1*_ Time 1 (baseline), *T*_*2*_ Time 2 (three months after baseline), *T*_*3*_ Time 3 (six months after baseline), *T*_*4*_ Time 4 (nine months after baseline)

**Table 4:** Fit indices of the cross-lagged models

	Cognitive self-stigma with Depression (Fig. 1)	Affective self-stigma with Depression (Fig. 2)	Behavioral self-stigma with Depression (Fig. 3)
χ^2^ (df)	17.81 (12)	38.96 (12)	2.19 (12)
p-value	0.12	< 0.001	0.99
CFI	0.996	0.981	0.999
NNFI	0.991	0.955	0.999
RFI	0.974	0.985	0.997
RMSEA	0.039	0.080	0.000
SRMR	0.035	0.046	0.042

*CFI* Comparative Fit Index, *NNFI* Non-Normed Fit Index, *RFI* Relative Fit Index, *RMSEA* Root Mean Square Error of Approximation, *SRMR* Standardized Root Mean Square Residual

Regarding the association between depressive features and cognitive self-stigma, Fig. 1 shows that baseline depressive features led to Time 2 cognitive self-stigma (coefficient = 0.37; *p* < 0.01); Time 2 cognitive self-stigma further led to Time 3 depressive features (coefficient = 0.09; *p* < 0.01); Time 3 depressive features subsequently led to Time 4 cognitive self-stigma (coefficient = 0.38; *p* < 0.01). A similar pattern was found in associations between depressive features and affective self-stigma. Figure 2 shows that baseline depressive features led to Time 2 affective self-stigma (coefficient = 0.31; *p* < 0.01); Time 2 affective self-stigma further led to Time 3 depressive features (coefficient = 0.10; *p* < 0.01); Time 3 depressive features subsequently led to Time 4 affective self-stigma (coefficient = 0.24; *p* < 0.01). A slightly different pattern was found in associations between depressive features and behavioral self-stigma. Figure 3 shows that baseline depressive features were not associated with Time 2 behavioral self-stigma, while Time 2 behavioral self-stigma led to Time 3 depressive features (coefficient = 0.11; *p* < 0.01) and Time 3 depressive features subsequently led to Time 4 behavioral self-stigma (coefficient = 0.15; *p* < 0.01).

However, given that the present sample consisted of mainly males, sensitivity analyses were used to examine if there were substantial differences between the results derived from the entire sample and those derived from the male sample. The sensitivity analysis results indicated that the results derived from males were comparable to those generated from analyses of the entire sample (Supplementary Tables S1 to S4; Figures S1 to S3).

## Discussion

The aim of the current study was to disentangle temporal associations between severities of depressive features and different types of self-stigma in a prospective cohort of people with SUDs. There were significant cross-sectional correlations between depressive features and all assessed forms of self-stigma. However, the cross-lagged models suggested that the directions of associations differed somewhat across different time periods and forms of self-stigma. Moreover, stronger associations between depressive features and subsequent self-stigma suggest that depressive features may promote self-stigma, particularly cognitive and affective forms. Implications are discussed below.

Only few prior studies have investigated associations between depressive features and self-stigma in people with SUDs. Wang et al. assessed how depression-related stigma may relate to alcohol and substance use [15]. They recruited 218 participants using a cross-sectional study and resonating with our findings observed that stigma related to depression was positively associated with mood disturbances and greater tendencies to use alcohol to cope with depression. According to the results, they suggested that stigma may be considered as a contributor for substance use in people with depression [15]. The positive association that we found in the current study between depressive features and self-stigma also may provide an explanation regarding how such an association may lead to excessive or problematic substance use as a compensatory solution to overcome depression.

Zeng et al. [43] investigated possible mechanistic roles of substance use and depressive features in association between stigma and suicidal behaviors in a group of migrant workers. They found that people with co-occurring depression and substance use had stronger levels of stigmatization, which may intensify their suicidal behaviors [43]. These findings are consistent with our findings of associations between depressive features and self-stigma in people with SUDs. Thus, we speculate that people with SUDs and high levels of self-stigma may be at high risk of harmful behaviors, such as suicide, and this possibility warrants direct examination. Accordingly, such individuals should receive further attention from healthcare systems and possibly be prioritized as a potentially high-risk and vulnerable population for treatment of depressive features, and reducing their self-stigma could be one potential target/benefit of such treatment. Additionally, directly targeting self-stigma through novel innovations warrants consideration.

Although as expected the direction of depressive features to self-stigma over time appeared stronger than the opposite pathway, the strength may have differed with respect to specific kinds of self-stigma. Overall, the cross-lagged associations between depressive features and cognitive and affective self-stigma appeared stronger than those observed between depressive features and behavioral stigma. Several possible explanations warrant consideration. First, many people with depressive symptoms are in initial phases of self-stigma, and self-stigma may not be reflected in their behaviors. Speculatively, they may prevent themselves from manifesting their beliefs and awareness and maintain self-stigma internally without public awareness. The findings also suggest these individual may be trying to cope with or resolve self-stigma without letting others know about it. A second non-mutually exclusive possibility may involve depressive features’ fluctuations over time and that resolution of depressive features may prevent escalation of self-stigma processes, as suggested by Corrigan et al. [27]. In fact, by reducing depressive features, the progression of self-stigma may be slowed or halted and associations between depressive features and behavioral self-stigma diminished or avoided.

Another point that should be addressed in the stronger temporal association between affective self-stigma and depression than other kinds of self-stigma is the formation process of such self-stigma. It is important to understand better how depressive features in people with SUDs may lead to the formation of negative attitudes and beliefs towards themselves. According to the self-concept theory, when people believe in differences between themselves and others regarding negative aspects of their nature (affective self-sigma), this may directly impact their communication with others, lead to lower self-esteem and sociability, and generate feelings of helplessness, consistent with depressive symptoms [44]. Therefore, a practical solution may exist for preventing the onset of stigmatization processes by identifying individuals and providing early treatment of depression, while the opposite but less significant direction also should be considered by controlling the negative attitudes that also may postpone depressive features reciprocally.

As discussed, there was no significant temporal association between behavioral self-stigma and depressive symptomology. This finding may be explained through the behavioral characteristics attributable to depressed mood. One symptom in people with depressed mood is social avoidance that may diminish the overt behavioral responses toward stimulations [45]. Therefore, because we observed reduced social interaction, behaviors related to stigmatization also may fade, and this may weaken associations between these variables, as found here. Furthermore, as noted in prior studies, behavioral responses in people with SUDs may be distorted [46, 47] in ways that may in part explain the absence of significant associations between these variables with depressed mood.

To the best of our knowledge, this is the first longitudinal study assessing temporal associations between depressive features and self-stigma in people with SUDs. Nonetheless, the current study included several limitations that should be mentioned. First, we used an accessible and convenient sample of people with SUDs who were admitted to a psychiatric center in Taiwan. Therefore, they were not representative of all Taiwanese people with SUDs or those from other regions and societies. As such, the extent to which the findings may generalize to other groups warrants direct examination. Second, we only used self-report scales to examine depressive symptoms. As such, future studies using clinical evaluations are required. Third, we followed participants for nine months and did not assess or control for multiple potential confounding factors during this period such as receiving treatment for depression. Therefore, it is recommended that future studies control for such variables and consider longer durations of assessment to better understand how depressive features and self-stigma may relate over time in people with SUDs. Finally, participants may have received different treatments according to their diagnoses and for different amounts of time. Therefore, the different treatments and amounts of time in treatment may have potentially confounded the present findings.

## Conclusion

The present study showed that depressive features and self-stigma are correlated positively in people with SUDs, and the relationships from depressive features towards promoting subsequent self-stigma appeared stronger than those in the opposite direction. These findings suggest the relevance of addressing depressive features in people with SUDs in order to prevent the generation of self-stigma in these people. In other words, the directional associations between depressive features and self-stigma suggest that any early diagnosis and treatment of depression among people with SUDs may prevent the subsequent development of self-stigma and thereby potentially protect such people from subsequent social isolation or related psychological distress. Further examination of temporal associations with more representative samples in other communities and cultures are suggested.

## Supplementary Information

**Additional file 1.**

**Additional file 2.**

**Additional file 3.**

**Additional file 4: Table S1.** Male participant characteristics (*N*=273). **Table S2.** Self-stigma anddepression across time among male participants. **Table S3.** Correlations betweenself -stigma and depression among male participants. **Table S4.** Fitindices of the cross-lagged models among male participants.

## Data Availability

The datasets generated during and/or analyzed during the current study are available from the corresponding author on reasonable request.

## Abbreviations

SUD: Substance use disorder
JPC: Jianan Psychiatric Center
IDCATPP: Integrated Demonstrative Center of Addiction Treatment Pilot Program
DASS-21-D: Depression, Anxiety, Stress Scale – Depression subscale
SSS-S: Self-Stigma Scale-Short
ANOVA: Analysis of variance
SEM: Structural equation modeling
CFI: Comparative fit index
NNFI: Non-normed fit index
RFI: Relative fit index
RMSEA: Root mean square error of approximation
SRMR: Standardized root mean square residual
