# Supplementary figures and images for: Temporal associations between depressive features and self-stigma in people with substance use disorders related to heroin, amphetamine, and alcohol use: a cross-lagged analysis
Source: BMC Psychiatry. 2022 Dec 21;22:815. doi: 10.1186/s12888-022-04468-z (PMC9768939; doi:10.1186/s12888-022-04468-z)

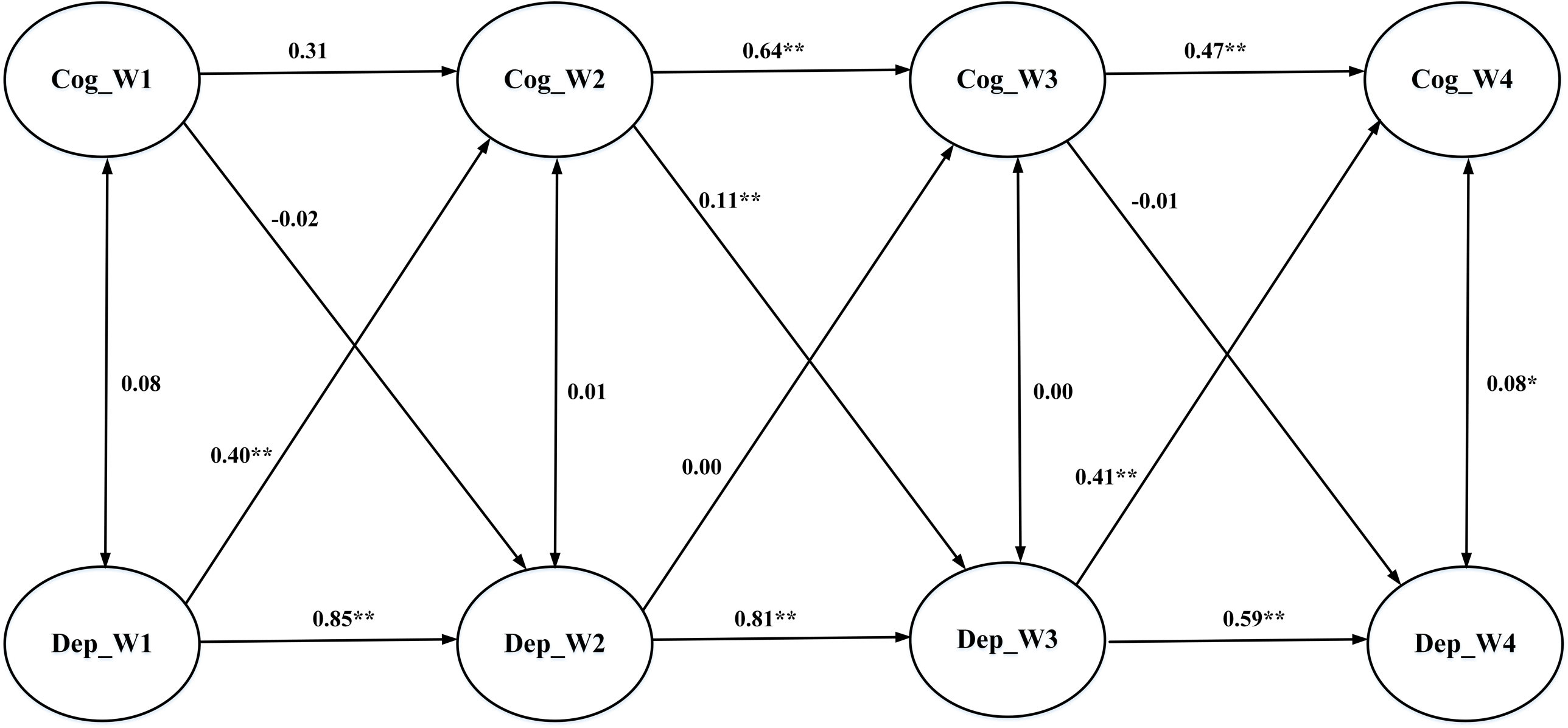

Supplement: Supplementary file 1 — Additional file 1. [file 12888_2022_4468_MOESM1_ESM.tif]

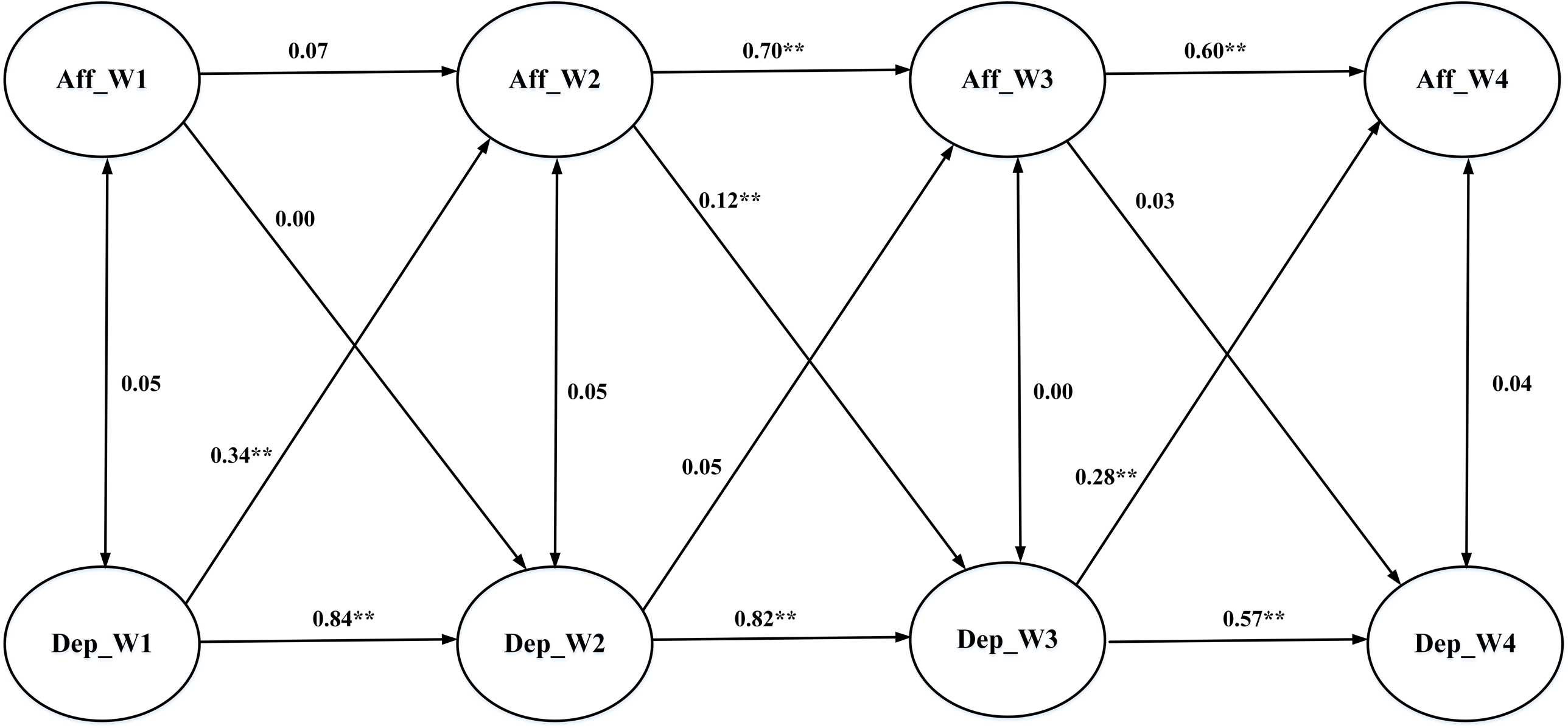

Supplement: Supplementary file 2 — Additional file 2. [file 12888_2022_4468_MOESM2_ESM.tif]

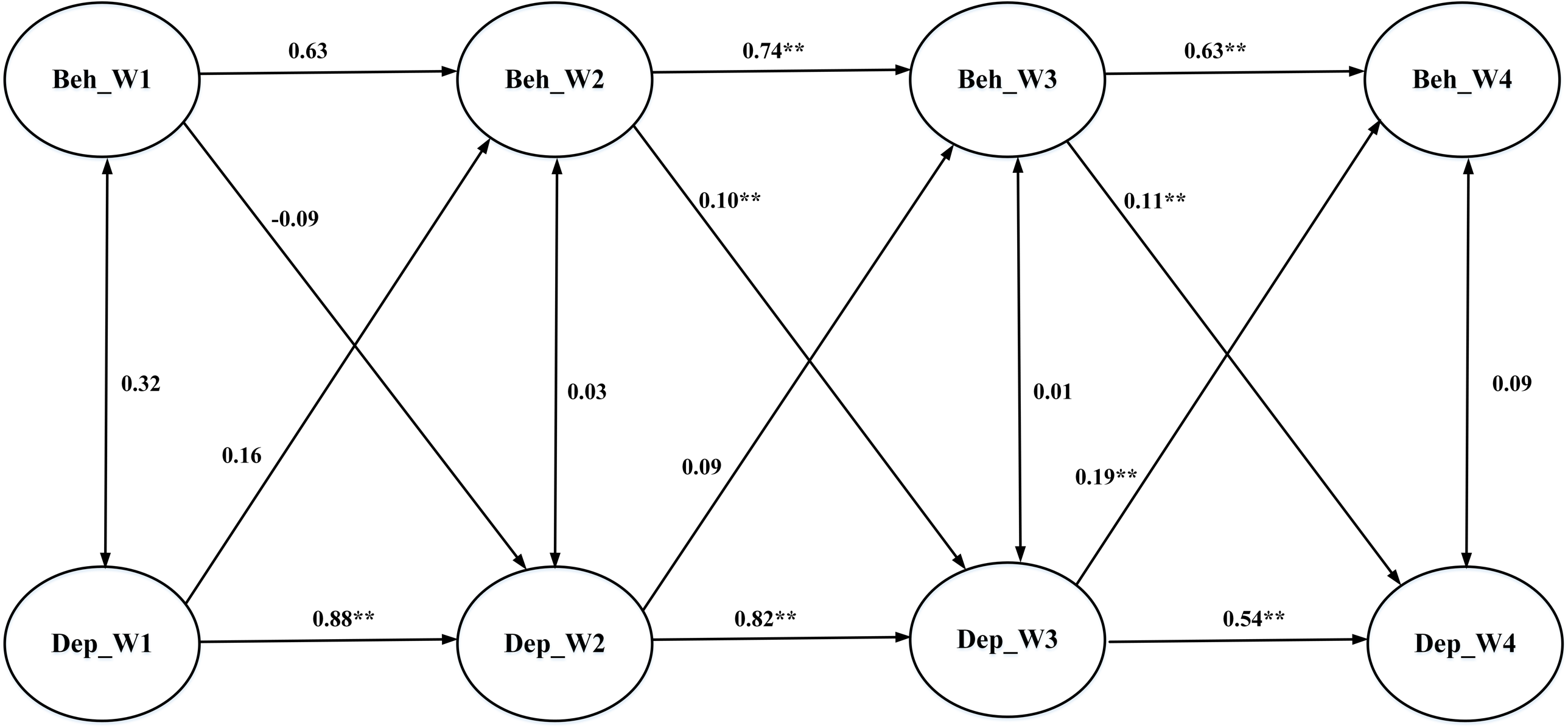

Supplement: Supplementary file 3 — Additional file 3. [file 12888_2022_4468_MOESM3_ESM.tif]
