# Supplementary material for: Temporal associations between depressive features and self-stigma in people with substance use disorders related to heroin, amphetamine, and alcohol use: a cross-lagged analysis
Source: BMC Psychiatry. 2022 Dec 21;22:815. doi: 10.1186/s12888-022-04468-z (PMC9768939; doi:10.1186/s12888-022-04468-z)
Supplement: Supplementary file 4 — Additional file 4: Table S1. Male participant characteristics (N=273). Table S2. Self-stigma anddepression across time among male participants. Table S3. Correlations betweenself -stigma and depression among male participants. Table S4. Fitindices of the cross-lagged models among male participants. [file 12888_2022_4468_MOESM4_ESM.docx]

Table S1 Male participant characteristics (N=273)

| Age (years); mean (SD) | 42.60 (9.10) |
| --- | --- |
| Marital status (single); n (%) | 148 (54.2%) |
| Education (junior high school degree or less); n (%) | 120 (44.0%) |
| Occupation (full time job); n (%) | 180 (65.9%) |
| Baseline depression ^a^; mean (SD) | 7.93 (10.51) |
| Self-Stigma Scale-Short Affect subscale; mean (SD) | 2.33 (0.81) |
| Self-Stigma Scale-Short Behavior subscale; mean (SD) | 2.69 (0.90) |
| Self-Stigma Scale-Short Cognitive subscale; mean (SD) | 2.21 (0.89) |

^a^ Multiplying the average score of Depression subscale in the Depression, Anxiety, Stress Scale (DASS-21) by 2.

Table S2. Self-stigma and depression across time among male participants

|  | Mean (SD) | | | | *F* (*p*-value) | Comparisons using Bonferroni adjustments |
| --- | --- | --- | --- | --- | --- | --- |
|  | Time 1 | Time 2 | Time 3 | Time 4 |  |  |
| Self-stigma_ Affect | 2.33 (0.81) | 2.15 (0.75) | 2.18 (0.79) | 2.18 (0.92) | 6.48 (*p*<0.01) | 1>2, 1>3, 1>4 |
| Self-stigma_ Behavior | 2.69 (0.90) | 2.49 (0.85) | 2.49 (0.87) | 2.36 (1.07) | 15.62 (*p*<0.01) | 1>2, 1>3, 1>4 |
| Self-stigma_ Cognition | 2.21 (0.88) | 2.05 (0.79) | 2.12 (0.75) | 2.09 (0.99) | 3.65 (*p*=0.02) | 1>2 |
| Depression | 7.92 (10.50) | 7.27 (10.00) | 7.25 (9.04) | 6.78 (8.38) | 1.82 (*p*=0.14) |  |

Self-stigma assessed using the Self-Stigma Scale-Short form; depression assessed using the Depression subscale of the Depression, Anxiety, Stress Scale (DASS-21). Time 1 =baseline; Time 2 = first follow-up (three months after baseline); Time 3 = second follow-up (six months after baseline); Time 4 = third follow-up (nine months after baseline); Post-hoc comparisons were Bonferroni-corrected.

Table S3. Correlations between self -stigma and depression among male participants

|  |  | r | | | | | | | | | | | | | |  |
| --- | --- | --- | --- | --- | --- | --- | --- | --- | --- | --- | --- | --- | --- | --- | --- | --- |
|  | 1 | 2 | 3 | 4 | 5 | 6 | 7 | 8 | 9 | 10 | 11 | 12 | 13 | 14 | 15 | 16 |
| 1. Self-stigma_ Affect_T_1_ | 1 |  |  |  |  |  |  |  |  |  |  |  |  |  |  |  |
| 1. Self-stigma_ Behavior_T_1_ | .80 | 1 |  |  |  |  |  |  |  |  |  |  |  |  |  |  |
| 1. Self-stigma_ Cognition_T_1_ | .81 | .73 | 1 |  |  |  |  |  |  |  |  |  |  |  |  |  |
| 1. Depression_T_1_ | .43 | .36 | .43 | 1 |  |  |  |  |  |  |  |  |  |  |  |  |
| 1. Self-stigma_ Affect_T_2_ | .64 | .65 | .58 | .32 | 1 |  |  |  |  |  |  |  |  |  |  |  |
| 1. Self-stigma_ Behavior_T_2_ | .63 | .68 | .54 | .34 | .86 | 1 |  |  |  |  |  |  |  |  |  |  |
| 1. Self-stigma_ Cognition_T_2_ | .58 | .59 | .62 | .39 | .85 | .80 | 1 |  |  |  |  |  |  |  |  |  |
| 8. Depression_T_2_ | .36 | .25 | .34 | .82 | .25 | .27 | .37 | 1 |  |  |  |  |  |  |  |  |
| 9. Self-stigma_ Affect_T_3_ | .47 | .42 | .49 | .27 | .61 | .60 | .55 | .18 | 1 |  |  |  |  |  |  |  |
| 10. Self-stigma_ Behavior_T_3_ | .57 | .58 | .50 | .33 | .66 | .68 | .57 | .25 | .85 | 1 |  |  |  |  |  |  |
| 11.Self-stigma_ Cognition_T_3_ | .42 | .41 | .52 | .29 | .61 | .56 | .61 | .20 | .85 | .78 | 1 |  |  |  |  |  |
| 12.Depression_T_3_ | .34 | .16 | .30 | .70 | .27 | .26 | .32 | .80 | .30 | .35 | .28 | 1 |  |  |  |  |
| 13.Self-stigma_ Affect_T_4_ | .56 | .50 | .44 | .45 | .61 | .58 | .55 | .38 | .55 | .63 | .51 | .38 | 1 |  |  |  |
| 14.Self-stigma_ Behavior_T_4_ | .54 | .56 | .43 | .40 | .59 | .60 | .54 | .35 | .50 | .60 | .46 | .35 | .92 | 1 |  |  |
| 15.Self-stigma_ Cognition_T_4_ | .46 | .32 | .45 | .51 | .44 | .44 | .50 | .44 | .50 | .51 | .54 | .46 | .85 | .78 | 1 |  |
| 16.Depression_T_4_ | .24 | .14^a^ | .12^b^ | .43 | .24 | .29 | .30 | .49 | .14^a^ | .22 | .12^b^ | .55 | .23 | .19 | .23 | 1 |

All p-values < 0.01, except for those with a subscript a (*p* = 0.02) or b (*p* = 0.04).

T_1_ = Time 1 (baseline); T_2_ = Time 2 (three months after baseline); T_3_ = Time 3 (six months after baseline); T_4_ = Time 4 (nine months after baseline)

Table S4. Fit indices of the cross-lagged models among male participants

|  | Cognitive self-stigma with Depression (Figure 1) |  | Affective self-stigma with Depression (Figure 2) | Behavioral self-stigma with Depression (Figure 3) |
| --- | --- | --- | --- | --- |
| χ^2^ (df) | 15.93 (12) |  | 26.20 (12) | 2.52 (12) |
| p-value | 0.19 |  | 0.01 | 0.99 |
| CFI | 0.997 |  | 0.989 | 0.999 |
| NNFI | 0.993 |  | 0.973 | 0.999 |
| RFI | 0.973 |  | 0.952 | 0.996 |
| RMSEA | 0.035 |  | 0.066 | 0.000 |
| SRMR | 0.034 |  | 0.044 | 0.043 |

CFI = Comparative Fit Index; NNFI = Non-Normed Fit Index; RFI= Relative Fit Index, RMSEA = Root Mean Square Error of Approximation; SRMR = Standardized Root Mean Square Residual
